# 3D printed teeth for bridge preparation training: development and assessment in dental education

**DOI:** 10.1186/s12909-025-07849-6

**Published:** 2025-09-10

**Authors:** Michael del Hougne, Johannes Schrenker, Isabella Di Lorenzo, Christian Höhne, Marc Schmitter

**Affiliations:** Department of Prosthodontics, University of Würzburg, Pleicherwall 2, 97070 Würzburg, Germany

**Keywords:** 3D printing, Dental education, Bridge preparation, Integrated workflow, Defect-oriented preparation technique, Additive manufacturing

## Abstract

**Background:**

Bridge preparation skills are a vital component of dental education and require specific techniques. This study aimed to develop and evaluate 3D printed teeth for use in defect-oriented bridge preparation and pre-prosthetic exercises in dental training, addressing the limited customization and lack of integrated workflows found in commercial typodont teeth. The null hypothesis stated that 3D printed teeth offered no advantage over established typodont training methods for bridge preparation.

**Methods:**

Custom 3D printed teeth were designed to support integrated workflows with defect-oriented preparation techniques and evaluated by fourth-year dental students in three sequential hands-on courses. Students had completed preclinical studies. Feedback was collected using a structured questionnaire comparing 3D printed teeth with conventional typodonts. Evaluation domains included pre-prosthetic exercise, preparation experience, learning process, learning success, and overall training, measured via Visual Analog Scales. Free-text responses captured perceived strengths and areas for improvement. Exercise complexity was reduced due to external factors and poor initial evaluations: pre-prosthetic exercises were omitted after the first course, and provisional bridge fabrication after the second.

**Results:**

A total of 116 fourth-year students participated. Although student evaluations were subjective, they provided valuable insight into the usability and learning experience of the 3D printed teeth. 3D printed teeth were rated relatively low in perceived realism in caries excavation (mean 30.58 ± 24.74) and core build-up placement (mean 47.77 ± 26.22). Tactile feedback during preparation was rated comparably (*p* = 0.906) for 3D printed and typodont teeth. Ratings for 3D printed teeth improved significantly from the first to third courses in tactile feedback (*p* = 0.008), learning process (*p* < 0.001), learning success (*p* < 0.001), and suitability (*p* < 0.001). Interest in using 3D printed teeth increased (*p* < 0.001), though students were cautious about fully replacing typodonts. Internal consistency was high (Cronbach’s α = 0.772–0.857). Free-text feedback highlighted the need for greater hardness (*n* = 43). Cost-effectiveness (*n* = 43) and realism (*n* = 18) were identified as key advantages.

**Conclusions:**

The 3D printed teeth allowed students to practice defect-oriented bridge preparation. Despite subjective data and methodological limitations, they show promise as a tool to support dental education.

## Background

Preparing teeth for bridge treatment is a routine procedure in dentistry. Frequently, the abutment teeth are compromised and require caries removal and core build-up restorations before initiating the actual tooth preparation. The resulting preparation techniques are technically demanding. Defect-oriented preparation techniques are required and involve customizing the tooth preparation to address specific defects or damage. This includes covering core build-ups and placing the preparation margin within healthy tooth structure, with the overall goal of preserving as much healthy tissue as possible while effectively restoring the damaged areas. Especially for endodontically treated teeth a “Ferrule Design” with 1.5 to 2 mm of healthy dentin is a predominant factor regarding survival [1, 2]. The invasiveness and extent of tooth structure removal varies upon preparation technique and material requirements [3].

Simulation-based training is an essential component of dental education, serving to prepare students for clinical practice in a controlled and structured environment. In German dental education, fourth-year students begin treating real patients under supervision, following extensive hands-on training and simulation exercises in the preceding years. In simulation exercises, commercially available typodont teeth (KaVo or Frasaco) are typically utilized. However, typodont teeth offer only limited scope for customization and scarcely permit an integrated workflow encompassing caries excavation, core build-up, and subsequent defect-oriented preparation. These limiting circumstances may contribute to challenges students face when transitioning to clinical settings, as observed during course supervision. Jazzar et al. reported that moving from preclinical to clinical training increases workload and anxiety among students, highlighting the importance of better integrating theoretical knowledge with practical skills and suggesting that curricula should be adapted to meet these challenges [4]. This demonstrated a need for targeted measures to enhance the quality of teaching.

3D printing is an advanced manufacturing technology that is rapidly evolving and has become widely adopted in the field of dentistry [5]. Tomášik et al. identified that 3D printing is the second most extensively researched area in orthodontics, following artificial intelligence [6]. 3D printing uses computer-aided design digital models to automatically produce customized 3D objects [7]. In their narrative review, Lepišová et al. discussed the wide range of applications, including the domains of restorative dentistry, regenerative dentistry and tissue engineering, as well as aspects such as the fabrication of oral guides [8]. In a retrospective cohort study involving 3D printed temporary crowns, a satisfactory survival rate of 98% was reported after 19 months of observation [9].

The integration of 3D printing into education has already been examined in several studies, highlighting its potential to enhance creativity, spatial thinking, and hands-on learning. Clear educational advantages have been associated with the integration of 3D printed tooth models into preclinical dental training. Duan et al. evaluated 3D printed teeth based on micro-CT data against traditional plastic models for preclinical access cavity preparation in senior undergraduates and over 80% of students supported incorporating 3D printed teeth into routine training. They concluded that 3D printed teeth could have the potential to replace conventional plastic teeth in preclinical training for access cavity preparation [10]. Di Lorenzo et al. reported that a 3D printed tooth for endodontic training, based on micro-CT data from an extracted natural tooth, proved to be beneficial. Its suitability as a training model and its handling were rated significantly higher than those of conventional acrylic blocks [11]. In their review, Karagkounaki et al. found that 3D printed dental models significantly enhance students’ clinical competence, confidence, and overall learning outcomes across various preclinical disciplines - including restorative, endodontic, prosthodontic, surgical, and pediatric simulation [12].

To address this educational gap and to provide a better preparation for students for their clinical training with patients, a set of 3D printed training teeth including a premolar and a molar was developed, utilized and modified for training of caries excavation, core build-up, and subsequent defect-oriented preparation for bridge treatment. The training teeth were used and evaluated subsequently across the academic terms by fourthyear students in a hands-on course (H) before they treated real patients.

Based on these considerations, the null hypothesis was formulated: the newly developed 3D printed teeth for training bridge preparation presented no advantage to the students compared to established training methods with typodont teeth.

Overall, the aims of this study were to develop and evaluate 3D printed teeth for use in defect-oriented bridge preparation and pre-prosthetic exercises in dental training.

## Materials and methods

The study and the experimental protocol were approved by the Institutional Review Board of the University of Würzburg (20181116 01) and the usage of irreversibly anonymized data from the questionnaire in the Department of Prosthodontics of the University of Würzburg was granted. All students that participated in the voluntary course were thoroughly informed and no personal data was collected. An informed consent to participate was obtained from all of the participants in the study. All methods were performed in accordance with the named guidelines, regulations and Declaration of Helsinki.

Fourth-year students participated in the hands-on courses. They had completed the preclinical phase of their studies and were about to begin their clinical training with patients.

### Design of 3D printed training teeth

KaVo-typodont teeth 14 and 16 were digitalized via the laboratory scanner InEos X5 (Dentsply Sirona) and processed with the software Blender (blender.org, Version 3.2). Processing included modelling the cavities and the corresponding base.

**Fig. 1:**
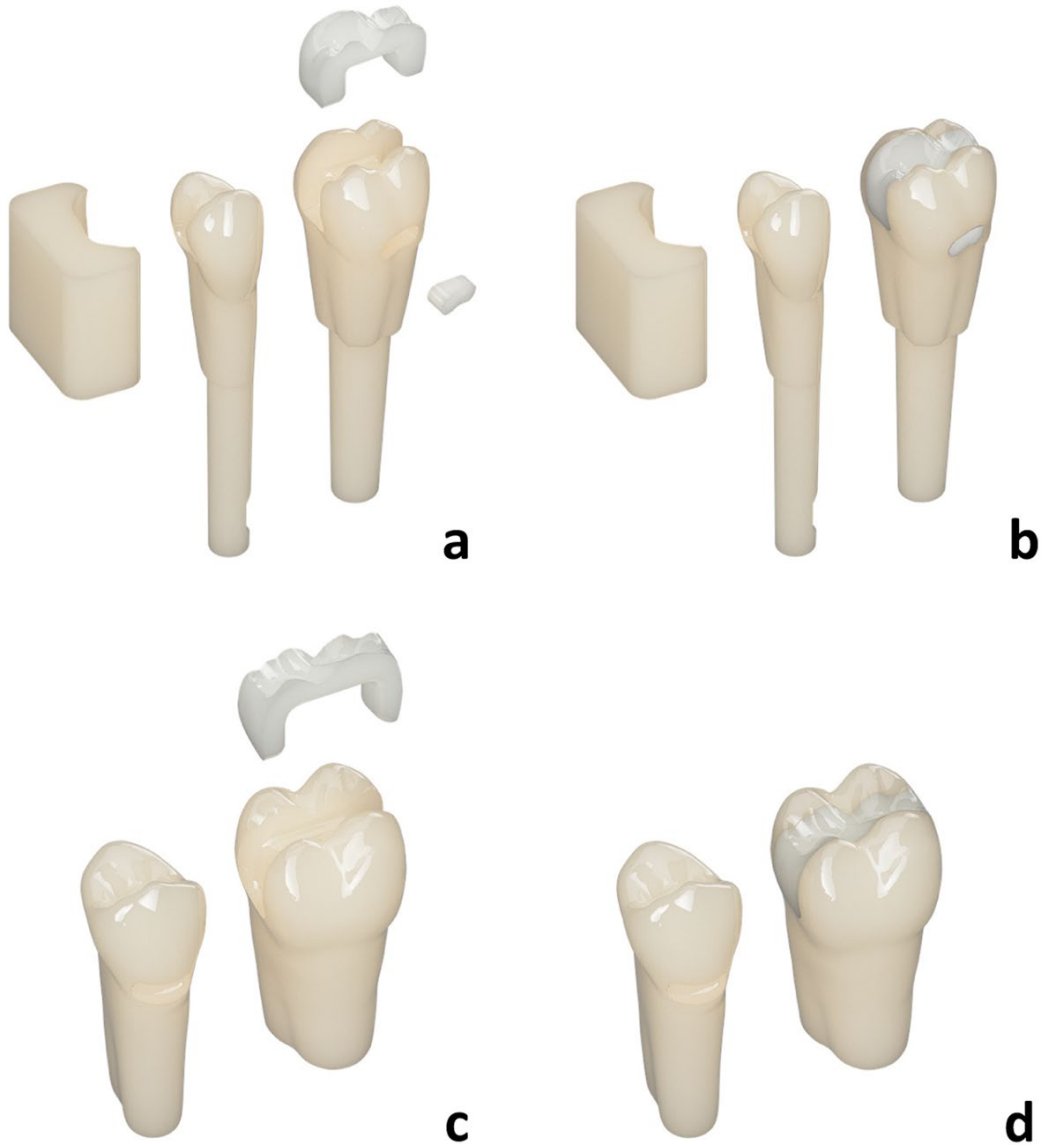
Schematic overview of designed training teeth. Only the digitally designed and 3D printed components are visualized, manually added components are not shown. (Source: own figure) (**a**) Premolar and molar for KaVo model from anterior-lateral with mold for premolar and offset core build-ups for molar. (**b**) Premolar and molar for KaVo model from anterior-lateral with mold for premolar. (**c**) Premolar and molar for Frasaco model from posterior-lateral with offset core build-ups for molar (**d**) Premolar and molar for Frasaco model from posterior-lateral

The first hands-on course (H1) had the most complex design of the training teeth, compared to the second (H2) and third (H3) hands-on course. The premolar 14 had a carious lesion and a cervical restoration and the molar 16 had a core build-up and a cervical restoration. A schematic overview of the training teeth of H1 is shown in Fig. 1a) and b). Both teeth were designed to fit into an upper jaw KaVo-typodont model to be mounted in a phantom head unit. A brief five-minute introductory presentation was provided to the students, outlining the objectives and procedural steps of the exercise. The materials to be used were presented; however, no visual aids or detailed explanations were given. At the beginning of the exercise, students created a mold using silicone putty for manufacturing a provisional restoration. Students then continued with the pre-prosthetic exercise and had to excavate the carious lesion of the premolar, followed by a core build-up with Rebilda DC White. Afterwards both teeth were prepared for a bridge restoration. The preparation exercise concluded with the fabrication of a provisional bridge restoration using ProTemp 4 (3 M ESPE AG, Germany). The silicone mold was trimmed, the prepared teeth were isolated with Vaseline, and the provisional restoration was then finished.

For the second (H2) and third (H3) hands-on course, the design of the training teeth was altered, resulting in a noticeable design drift from the original model used in H1. These changes were prompted by external factors including time constraints caused by updates to the dental licensing regulations and the unfavorable evaluations of the 3D printed training teeth in H1. Consequently, the exercise was condensed and simplified. Students were given a 10-minute presentation in advance of the hands-on course showcasing the required preparation technique, stating the necessity of fully covering core build-ups and placing preparation margin in healthy tooth structure and presenting desired preparation outcome.

The design modifications resulted in the removal of the carious lesion on the premolar and the cervical restoration on the molar. Additionally, the otherwise identical training teeth were redesigned to be compatible with an upper jaw Frasaco typodont model for mounting in a phantom head unit. Due to the changes in the dental licensing regulations, students had access only to Frasaco models—rather than both KaVo and Frasaco models as in previous preclinical education. A schematic overview of the training teeth of H2 and H3 is displayed in Fig. 1c) and d). The step involving the fabrication of provisional restorations was excluded from H3.

An overview of the scope of the introductory presentation, the workflow, and the model for mounting the 3D printed teeth is presented in Table 1.

**Table 1:** Scope of introductory presentation, workflow and models utilized for mounting the 3D printed teeth for hands-on courses H1, H2 and H3

	Introductory presentation	Workflow	Model for mounting 3D printed teeth
H1	5 min	- Creation of mold with silicone for provisional restoration	KaVo
		- Excavation of carious lesion	
		- Core build-up	
		- Preparation for bridge restoration	
		- Fabrication of provisional bridge restoration	
H2	10 min	- Creation of mold with silicone for provisional restoration	Frasaco
		- Preparation for bridge restoration	
		- Fabrication of provisional bridge restoration	
H3	10 min	- Preparation for bridge restoration	Frasaco

### Fabrication of 3D printed training teeth

**Fig. 2:**
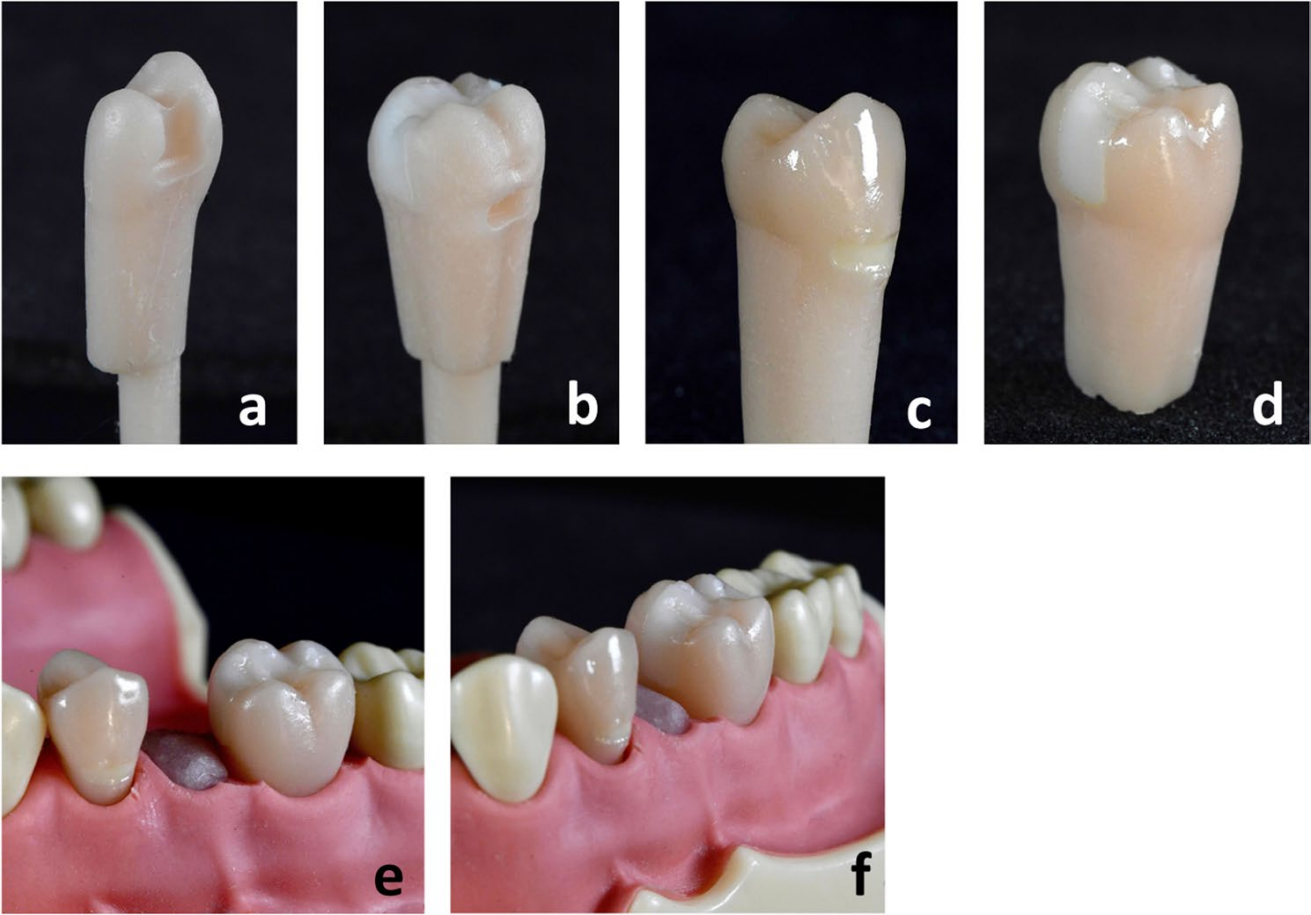
3D printed training teeth. (Source: own figure) (**a**) Premolar for KaVo model without Telio Onlay in cavity and without varnish. (**b**) Molar for KaVo model without cervical restoration and without varnish. (**c**) Premolar for Frasaco model with cervical restoration and varnish. (**d**) Molar for Frasaco model with core build-up and varnish. (**e**) 3D printed teeth in Frasaco model, view from lateral. (**f**) 3D printed teeth in Frasaco model, view from anterior

A Form 3B (Formlabs Inc.) 3D printer was utilized to produce the training teeth. They were 3D printed with Model V3 Resin (RS-F2-DMBE-03, Formlabs Inc.), which is generally utilized for the production of high-accuracy restorative models. The core build-up of the molar was printed with Rigid 4 K Resin (RS-F2-RGWH-01, Formlabs Inc.), a glass-filled resin with high precision and stiffness. Model V3 Resin was utilized to assemble the core build-up into the molars. Post-processing steps, including washing and curing (Form Wash & Form Cure, Formlabs Inc.), were carried out in accordance with the manufacturer’s recommendations. In preparation of the first hands-on course, each carious lesion in the premolar was filled and light cured with the temporary resin Telio Onlay (Ivoclar Vivadent GmbH, Germany), which enables the simulation of caries excavation with burs. The cervical cavities were filled with Rebilda DC White (Voco GmbH, Germany) and light cured. Finally, a thin layer of the light-curable, single-component varnish (Luxatemp Glaze & Bond, DMG Chemisch-Pharmazeutische Fabrik GmbH, Germany) was applied to the teeth and polymerized using a curing light. This resulted in a glossy surface finish, contributing to a more natural esthetic. Figure 2 showcases the finished 3D printed teeth.

### Questionnaire for evaluation

A questionnaire, as shown in Table 2, was developed utilizing Eva-Sys (Electric Paper Evaluationssysteme GmbH) under consultation of the Institute for Clinical Epidemiology and Biometry (IKE-B) at the Julius-Maximilians-University of Würzburg.

**Table 2:** Questionnaire

1	Pre-prosthetic exercise
1.1	How realistic do you consider the caries excavation on the 3D printed training teeth? VAS (very unrealistic – very realistic)
1.2	How realistic do you consider the placement of the core build-up on the 3D printed practice teeth? VAS (very unrealistic – very realistic)
2	Preparation
2.1	How do you assess the tactile feeling during the preparation of the 3D printed practice teeth? VAS (very unrealistic – very realistic)
2.2	How do you assess the tactile feeling during the preparation of KaVo/Frasaco typodont teeth? VAS (very unrealistic – very realistic)
3	Learning process
3.1	How do you assess the learning process during preparation exercises with 3D printed practice teeth? VAS (very bad – very good)
3.2	How do you assess the learning process during preparation exercises with KaVo/Frasaco teeth? VAS (very bad – very good)
4	Learning success
4.1	How do you assess the learning outcome of preparation exercises with 3D printed practice teeth? VAS (very bad – very good)
4.2	How do you assess the learning outcome of preparation exercises with KaVo/Frasaco typodont teeth? VAS (very bad – very good)
5	Assessment of the training
5.1	How do you assess the suitability of the 3D printed training teeth as preparation for patient treatment? VAS (very bad – very good)
5.2	How do you assess the suitability of the KaVo/Frasaco typodont teeth as preparation for patient treatment? VAS (very bad – very good)
5.3	For my studies, I would like to have more practice exercises with 3D printed training materials. VAS (totally disagree – totally agree)
5.4	I can imagine completing the entire training to prepare for patient treatment exclusively with 3D printed training materials. VAS (totally disagree – totally agree)
6	Free text questions
6.1	What could still be improved on the 3D printed practice teeth?
6.2	What advantages do you think 3D printed training materials offer in dental education?

Q1.1 and Q1.2 only included in H1

For the first hands-on course (H1), the questionnaire included the evaluation of pre-prosthetic exercise, preparation exercise, learning process, learning success and evaluation of training feasibility. The 3D printed training teeth were compared to KaVo typodont teeth. Visual Analog Scale (VAS) items were implemented for quantification. They included two opposing descriptives at each end of the 10 cm long line. No predefined cut-off values were applied to the VAS scores; instead, the scale was treated as a continuous measure to capture the full range of subjective responses.

For the second (H2) and third (H3) hands-on course the questionnaire was adapted; the domain regarding the pre-prosthetic exercise was removed (Sect. 1 in Table 2) and 3D printed training teeth were compared to Frasaco typodont teeth.

### Statistics

IBM SPSS Statistics (Version 29; IBM Corp., Armonk, NY, USA) and G*Power (Version 3.1.9.7) were used for statistical analyses. Descriptive statistics were computed, and the Shapiro-Wilk test assessed normality. For comparisons between two independent groups, the Mann-Whitney U test was applied. The Kruskal-Wallis test was used for comparisons among multiple independent groups. Statistical power was estimated via a one-way ANOVA (fixed effects, omnibus) model in G*Power, based on effect sizes converted from the Kruskal-Wallis results, providing an appropriate approximation for detecting group differences. Significant Kruskal–Wallis results were followed by pairwise post hoc comparisons using the Dunn test with Bonferroni correction. Effect sizes and statistical power were reported throughout. Cronbach’s alpha was determined for evaluation of internal consistency. The significance level was set at α = 0.05.

Two independent examiners manually grouped the responses to the free-text questions by identifying common themes and categorizing them accordingly. When discrepancies arose, they engaged in detailed discussions to reach a consensus, thereby ensuring consistency and reliability in the categorization process. This collaborative method helped uphold the validity of the thematic grouping.

## Results

Overall, 116 students participated in the three hands-on courses between April 2024 and April 2025 at the Department of Prosthodontics of the University of Würzburg with 41 participants in H1, 35 participants in H2 and 38 participants in H3.

### Results of the VAS-items

**Fig. 3:**
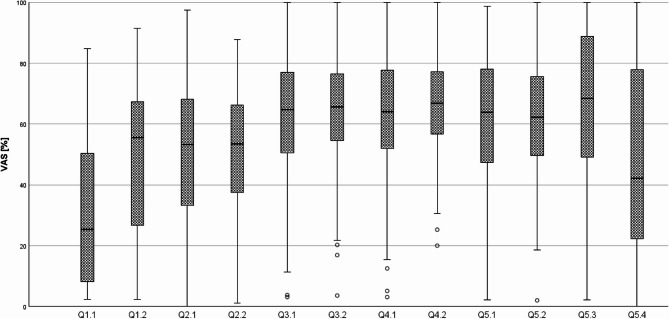
Boxplot of individual questions (Q) and VAS percentages – cumulated results of H1, H2 and H3. (Source: own figure)

**Fig. 4:**
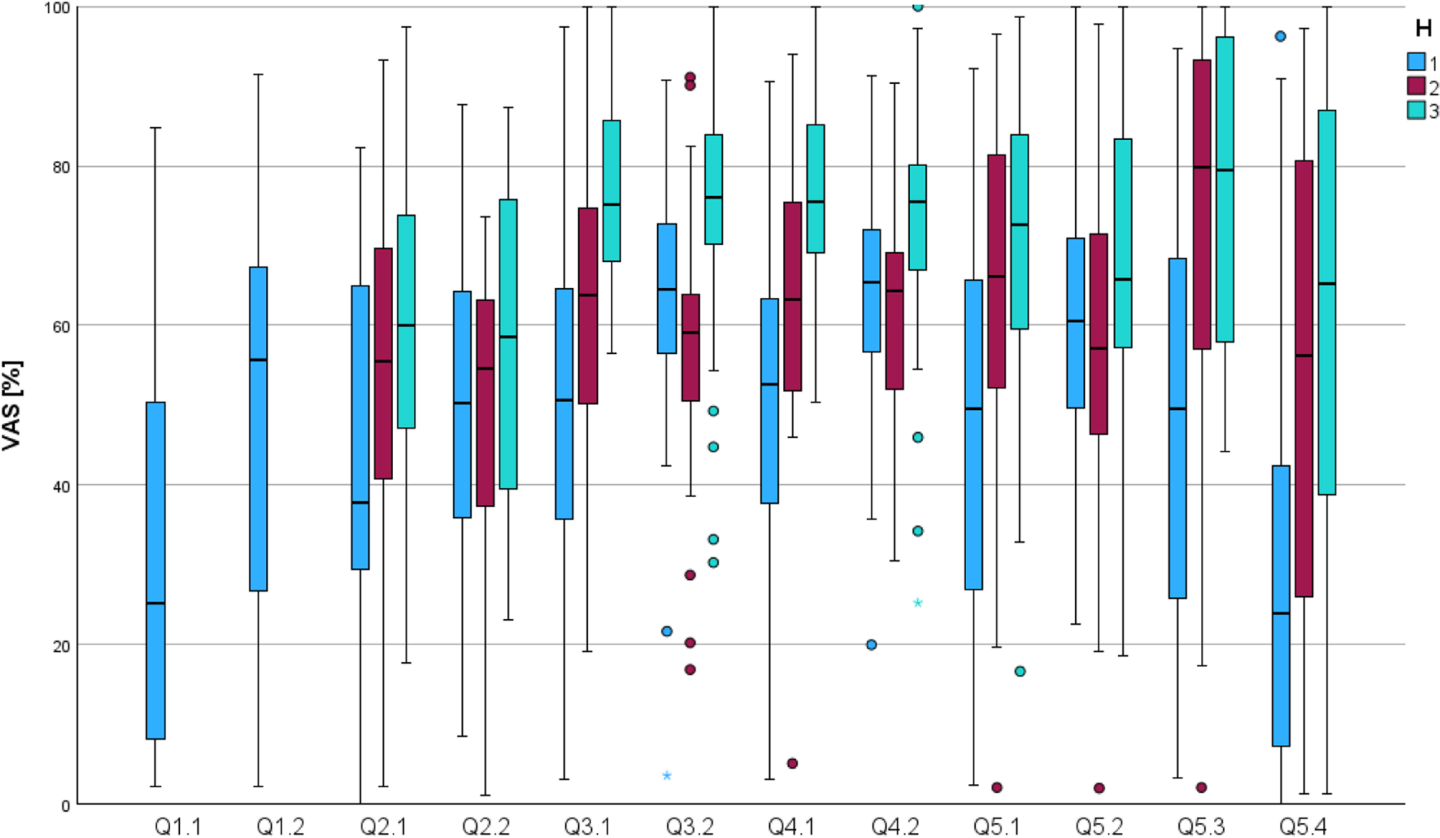
Boxplot of individual questions (Q) and VAS percentages - separated by groups H1, H2, and H3. (Source: own figure)

The VAS items had a 10 cm long line and the marked positions were measured and converted into percentages for each question (Q). Figure 3 displays the cumulative results as a boxplot, while Fig. 4 shows the distributions separated by H1, H2, and H3. An overview of cumulative and individual results from H1, H2 and H3 with values for mean and standard deviation for each question is provided in Table 3.

**Table 3:** Overview of cumulative (total) and individual results and from H1, H2 and H3 with values for mean and standard deviation for each question

Question		Mean	Standard deviation
Q1.1	Total = H1	30.58	24.76
Q1.2	Total = H1	47.77	26.22
Q2.1	Total	51.14	22.30
	H1	42.48	21.89
	H2	53.80	22.27
	H3	58.15	20.08
Q2.2	Total	51.87	18.73
	H1	48.44	18.35
	H2	50.46	18.28
	H3	56.92	18.96
Q3.1	Total	62.48	21.37
	H1	50.50	23.06
	H2	61.61	18.64
	H3	76.61	11.61
Q3.2	Total	64.97	17.43
	H1	62.46	15.98
	H2	57.85	16.17
	H3	74.70	16.14
Q4.1	Total	63.67	20.03
	H1	51.31	20.73
	H2	64.37	16.81
	H3	76.68	12.58
Q4.2	Total	65.91	15.51
	H1	62.87	14.49
	H2	62.11	14.26
	H3	72.99	15.73
Q5.1	Total	60.67	24.38
	H1	47.84	24.90
	H2	64.89	23.00
	H3	71.07	18.49
Q5.2	Total	61.15	20.06
	H1	59.82	16.62
	H2	57.11	21.23
	H3	66.70	21.69
Q5.3	Total	64.81	26.37
	H1	47.62	25.05
	H2	72.86	24.94
	H3	76.32	18.38
Q5.4	Total	47.84	31.72
	H1	33.44	29.58
	H2	50.36	31.73
	H3	61.73	27.66

The statistics included pairwise comparisons using the Mann–Whitney U test to assess pairwise comparisons for the overall results of H1, H2 and H3. This analysis reported statistical significance (p), effect sizes (r), and statistical power (1-β); the results are summarized in Table 4. Across all comparisons, no statistically significant differences were found. The effect sizes were negligible, and statistical power was consistently low, indicating a limited ability to detect small effects.

**Table 4:** Results of pairwise comparisons of items for overall results of H1, H2 and H3 with Mann-Whitney U test. Statistical significance (p), effect sizes (r), and power (1 – β) are reported

	*p*	*r*	1-β
Q2.1-Q2.2	0.906	0.008	0.052
Q3.1-Q3.2	0.541	0.040	0.093
Q4.1-Q4.2	0.507	0.044	0.101
Q5.1-Q5.2	0.688	0.027	0.069

The pre-prosthetic exercise (Q1.1 and Q1.2) was evaluated exclusively within group H1. Thereby, the realism of caries excavation using the 3D printed training teeth was rated at a mean of ⌀30.58, while the placement of the core build-up received a mean rating of ⌀47.77. The tactile feedback during preparation (Q2.1 and Q2.2) was evaluated across all groups. Thereby 3D printed teeth received an overall mean rating of ⌀51.14, and the typodont teeth ⌀51.87. Participants rated the learning process (Q3.1 and Q3.2) comparably for both materials, with the 3D printed teeth achieving a mean score of ⌀62.48 and the typodont teeth ⌀64.97. Regarding perceived learning success (Q4.1 and Q4.2), the 3D printed teeth received a mean rating of ⌀63.67, and the typodont teeth ⌀65.91. In evaluating suitability for training (Q5.1 and Q5.2), ratings were comparable: ⌀60.67 for the 3D printed teeth and ⌀61.15 for the typodont teeth. When asked about their interest in more practice opportunities using 3D printed training materials (Q5.3), students responded positively, with a mean agreement score of ⌀64.81. However, in terms of fully replacing conventional materials with 3D printed alternatives throughout the training curriculum (Q5.4), students expressed reservations, reflected in a lower mean rating of ⌀47.84.

To evaluate overall differences between H1, H2 and H3, Kruskal–Wallis tests were applied for each item of the questionnaire. Statistical significances (p), effect sizes (ε²) and power estimates (1-β) are summarized in Table 5. No statistically significant group differences were revealed for Q2.2 and Q5.2.

**Table 5:** Results of Kruskal-Wallis test for H1, H2 and H3 for items of questionnaire. Statistical significance (p), effect sizes (ε2), and power (1 – β) are reported

	*p*	ε^2^	1-β
Q2.1	0.007	0.071	0.744
Q2.2	0.168	0.014	0.187
Q3.1	< 0.001	0.271	1.000
Q3.2	< 0.001	0.199	0.998
Q4.1	< 0.001	0.269	1.000
Q4.2	< 0.001	0.114	0.934
Q5.1	< 0.001	0.143	0.976
Q5.2	0.097	0.024	0.296
Q5.3	< 0.001	0.223	1.000

For items with statistically significant results in the Kruskal-Wallis test, post hoc pairwise comparisons were conducted using the Dunn test with bonferroni correction, further clarifying which specific group comparisons contributed to the observed differences. Table 6 reports statistical significances (p), effect sizes (r), and power (1-β).

**Table 6:** Results of Dunn-Test with bonferroni correction for pairwise comparisons between H1, H2 and H3 for each questionnaire item. Statistical significance (p), effect sizes (r), and power (1 – β) are reported

	*p*	*r*	1-β
Q2.1			
H1-H2	0.075	0.255	0.451
H1-H3	0.008	0.339	0.767
H2-H3	1	0.084	0.046
Q3.1			
H1-H2	0.11	0.238	0.387
H1-H3	< 0.001	0.637	1.000
H2-H3	0.002	0.399	0.894
Q3.2			
H1-H2	0.387	0.173	0.188
H1-H3	0.002	0.385	0.887
H2-H3	< 0.001	0.559	0.999
Q4.1			
H1-H2	0.034	0.289	0.579
H1-H3	< 0.001	0.639	1.000
H2-H3	0.008	0.350	0.769
Q4.2			
H1-H2	1	0.055	0.030
H1-H3	0.005	0.354	0.811
H2-H3	0.001	0.410	0.914
Q5.1			
H1-H2	0.008	0.341	0.765
H1-H3	< 0.001	0.457	0.978
H2-H3	0.976	0.116	0.078
Q5.3			
H1-H2	< 0.001	0.477	0.988
H1-H3	< 0.001	0.524	0.998
H2-H3	0.687	0.047	0.025
Q5.4			
H1-H2	0.05	0.273	0.517
H1-H3	< 0.001	0.463	0.982
H2-H3	0.319	0.190	0.217

The tactile feedback rating of the 3D printed teeth progressively increased from H1 to H2 and further to H3. It was rated significantly lower (*p* = 0.008) in H1 (⌀42.48) compared to H3 (⌀58.15).

The ratings of the learning process with 3D printed teeth increased from H1 to H2 and then to H3. Thereby, the ratings in H1 (⌀50.50) were significantly lower (*p* < 0.001) than in H3 (⌀76.61), and ratings in H2 (⌀61.61) were also significantly lower (*p* = 0.002) than in H3. The learning process with typodont teeth was rated lowest in H2 (⌀57.85). This was significantly lower (*p* < 0.001) than in H3 (⌀74.70), and in H1 (⌀62.46) also significantly lower (*p* = 0.002) compared to H3.

The ratings for learning success with 3D printed teeth increased from H1 to H2 and further to H3. In H3 (⌀76.68), the rating was significantly higher (*p* < 0.001) than in H1 (⌀51.31;), and the rating in H3 was also significantly higher (*p* = 0.008) than in H2 (⌀64.37). The learning success with typodont teeth was rated highest in H3 (⌀72.99). Thereby, ratings in H3 were significantly higher (*p* = 0.001) than in H2 (⌀62.11), and ratings in H3 were also significantly higher (*p* = 0.003) than in H1 (⌀62.87).

The suitability of the 3D printed teeth in preparation for patient treatment was rated lowest in H1 (⌀47.84). This was significantly lower (*p* = 0.008) than in H2(⌀57.11), and significantly lower (*p* < 0.001) than in H3(⌀71.07).

For the item assessing interest in further practice with 3D printed training materials, participants in H2 (⌀72.86) and H3 (⌀76.32) expressed significantly higher (both *p* < 0.001) agreement compared to those in H1 (⌀47.62).

Finally, when asked whether they could imagine completing the entire training using only 3D printed materials, students in H1 (⌀33.44) rated this option significantly lower (*p* < 0.001) than those in H3 (⌀61.73).

Cronbach’s alpha was calculated for H1 at α = 0.857, H2 at α = 0.781 and H3 at α = 0.772, representing good internal consistency.

### Results of the free text questions

For the free text questions (Q6.1 and Q6.2), similar items were grouped and counted for H1, H2, and H3 as well as added up, as shown in Table 7.

**Table 7:** Results of the free text questions (Q6.1 and Q6.2) for H1, H2, H3 and in total

	H1	H2	H3	total
Suggested improvements on the 3D printed practice teeth (Q6.1)				
increase hardness	15	7	21	43
provisional restoration difficult due to unintended adhesion with 3D printed teeth	3	3	0	6
enamel layer missing	2	0	3	5
improve bonding of components	0	2	3	5
improve retention in model	1	2	1	4
improve form of teeth	1	1	0	2
bad visibility of core build-up	1	1	0	2
caries excavation unrealistic	2	0	0	2
loss of definition at the preparation margin	1	0	1	2
unrealistic surface structure	0	2	0	2
implement pulp chamber	1	0	0	1
deeper core build-up	0	0	1	1
Advantages of 3D printed training materials in dental education (Q6.2)				
reduced costs	18	8	17	43
more realistic practice opportunity	4	7	7	18
individual tooth shape, position and defects possible	1	8	4	13
flexibility of the practice	4	4	4	12
patient realistic situation enabled	3	2	1	6
good and useful practice opportunity	0	5	0	5
realistic workflow with caries excavation and core build-up	4	0	0	4
better simulation of color than typodont teeth	1	3	0	4
better simulation of hardness than typodont teeth	2	1	0	3
diversion as a practice	1	2	0	3
enabling defect-oriented preparation	0	3	0	3
enables utilization of different materials	0	2	1	3
practice of difficult situations	1	0	0	1
training without risk to patients	1	0	0	1
availability	0	0	1	1
future proof	0	0	1	1
efficient	0	0	1	1
boost in self-confidence	0	0	1	1

### Cost of 3D printed training teeth

Pure resin costs for the 3D printed training teeth for H1 were 13.20€ for Model V3 Resin and 2.82€ for Rigid 4 K Resin. Due to the modified design, H2 required 8.71€ for Model V3 Resin and 1.66€ for Rigid 4 K Resin, while H3 required 9.65€ for Model V3 Resin and 1.80€ for Rigid 4 K Resin. In total, the resin material costs for all three hands-on courses amounted to €37.84.

Additional costs included resin for manual assembly of the teeth, isopropanol alcohol for washing and Rebilda DC White for the cervical cavities. Furthermore, for the first hands-on printing the mold and Telio Onlay for the carious lesion were required.

## Discussion

The 3D printed training teeth for bridge preparation training in H1 provided the most comprehensive simulation of an integrated clinical workflow within this study - including caries excavation, core build-up, prosthetic preparation, and the fabrication of provisional restorations - the subsequent training sessions focused more specifically on bridge preparation and the essential techniques for fully covering core build-ups with preparation margins placed in sound tooth structure. This led to a stepwise reduction in task complexity from H1 to H2 and further to H3. Therefore, limitations and methodological considerations must be addressed, along with a discussion of student feedback and an analysis of the educational implications.

### Limitations and methodological considerations

Several methodological limitations should be acknowledged when interpreting the results of this study. First, the absence of randomization, crossover design, and examiner blinding may have introduced potential biases. Additionally, commercially available typodont teeth were used solely as a comparative reference rather than employing an objective benchmark, such as extracted human teeth. These constraints reflect the applied, curriculum-integrated context of the study and should be addressed in future controlled trials with more rigorous experimental designs.

Furthermore, the outcomes in this study were based exclusively on subjective assessments using VAS, capturing students’ perceptions rather than objective performance metrics. While VAS ratings offer valuable insights into student experience and engagement, they are inherently limited by individual variability and expectation. This is particularly relevant in the context of fourth-year dental students, whose clinical experience with real carious lesions and complex restorative procedures is limited. Their ability to assess realism in areas such as caries excavation or core build-up placement may therefore be constrained, and interpretations of realism should be viewed with caution.

The nature of item Q5.3 may also be seen as leading or suggestive, potentially biasing responses in favor of positive engagement with the new materials. Future questionnaire designs should ensure more neutral phrasing to avoid response bias.

Statistical analyses were primarily exploratory. Although post hoc corrections were applied to pairwise comparisons, no correction for multiple testing across all VAS items was implemented. While this approach allowed for a broad exploratory understanding, it increases the risk of Type I errors. Future studies may benefit from multivariate statistical models to address multiplicity, interaction effects, and longitudinal comparisons.

Finally, the progressive simplification of the training exercises from H1 to H3 - driven by external factors such as regulatory changes - reduced the complexity of tasks and limited comparability across sessions. While simplification improved student evaluations, it also confounded direct interpretation of training effectiveness over time. This highlights the need for consistent exercise structure and comparable workload across evaluation periods to ensure valid comparisons.

### Student feedback

Many participants (*n* = 43) recommended increasing the hardness of the 3D printed teeth. Related aspects were a loss of definition at the preparation margin (*n* = 2) and an unrealistic surface structure (*n* = 2). These aspects may negatively impact the learning outcome. Inadequate hardness can lead to unrealistic tactile feedback, impairing the development of proper pressure control and hand skills. Moreover, indistinct margins hinder the ability to practice precise preparation techniques essential for clinical success. These factors may compromise the transfer of learned skills to real patient care, highlighting the need for improved material properties. The material used in the present study was Model V3 resin, which, after post-curing, exhibits a flexural modulus of 2.2 GPa, according to manufacturer specifications [13]. In comparison, the flexural modulus of root dentin has been reported as 17.5 ± 3.8 GPa [14], indicating that the mechanical stiffness of the 3D printed training teeth is considerably lower. Additionally, students noted the absence of an enamel layer in the printed teeth, highlighting the challenges in replicating enamel realistically using current 3D printing resins. An alternative material, the Rigid 10 K resin (Formlabs Inc.), may enhance the mechanical properties of the printed teeth due to its high stiffness and hard texture, which is reinforced with glass fibers. Formlabs Inc. reports in their technical data sheet a flexural modulus of 10 GPa for this resin following appropriate post-curing [15]. However, its greyish to beige coloration may detract from the esthetic quality of the training models. This represents a disadvantage, as students reported (*n* = 2) on bad visibility of the core build-up for the materials with comparatively better esthetics properties. Insufficient bonding of the components of the 3D printed teeth was reported (*n* = 5) and could be improved by enhanced post-curing or utilization of specific dental adhesion materials. Cresswell-Boyes et al. concluded that in their study with their specific setup, 3D printed teeth provided haptic feedback comparable to that of extracted human teeth and represented a valuable tool for preclinical dental education – when compared to commercially available typodont teeth, 3D printed teeth offered superior tactile realism, enhancing the simulation of clinical procedures [16]. Höhne et al. reported a 3D printed modular training model based on a patient’s cone beam computed tomography, incorporating enamel and dentin layers that provided both a realistic optical appearance and appropriate tactile feedback during preparation [17].

Students highlighted that the 3D printed teeth offered realistic practice opportunities (*n* = 18), along with anatomically accurate tooth shapes, positions, and defect representations (*n* = 13). The flexibility of the exercises (*n* = 12) and the simulation of patient-like clinical scenarios (*n* = 6) were also positively noted. These findings align with previous studies demonstrating that 3D printed models based on real patient data, as well as purpose-designed training models in pediatric dentistry, can significantly enhance the authenticity and educational value of hands-on training [18, 19]. The student feedback underscores the potential of 3D printing to bridge the gap between traditional typodont-based simulations and authentic clinical experiences. Unlike conventional mass-produced plastic teeth, which often lack anatomical variation and clinical complexity, 3D printed models offer a high degree of customization. In the context of the present study, this included the integration of features, such as carious lesions and core build-ups, that more accurately reflect real-world restorative challenges.

An improved retention of the 3D printed teeth was desired (*n* = 4) for both the KaVo and Frasaco models. The KaVo model utilizes a click mechanism for tooth retention, whereas the Frasaco model employs screws. Designing 3D printed teeth to fit the KaVo system proved challenging and labor-intensive, achieving only limited success. Conversely, the material properties of the 3D printed teeth in the Frasaco model complicated secure screwing, as the screws were prone to over-tightening, resulting in inadequate retention.

Students in H1 (*n* = 3) and H2(*n* = 3) reported difficulties in fabricating the provisional bridge restorations. These challenges were primarily due to unintended adhesion between the provisional material and the surface of the 3D printed teeth, even when Vaseline was used as a separating medium. This adhesion complicated removal and finishing of the provisional restorations. Students had prior experience fabricating provisional restorations on typodont teeth and understood the necessity of isolation using Vaseline. Lubrication with petroleum jelly or a suitable separating media represents a standard protocol in fabricating provisional restorations [20]. In the context of 3D printed materials, surface energy and texture may promote interaction with resin-based materials, even when lubricated. Surface treatments to optimize bond strength and manage adhesion behavior with 3D printed resins have been demonstrated in both fresh and aged materials [21, 22]. Therefore, implementing appropriate surface conditioning protocols is critical to balance the need for adequate provisional restoration retention with the requirement for reliable and clean removal.

In H1, students (*n* = 2) reported that the caries excavation appeared unrealistic. The carious lesion model used with Telio Onlay is well-established and familiar to the students; therefore, criticism of this exercise was not anticipated. Carnier et al. demonstrated that more realistic caries simulation can be achieved using 3D printed teeth with artificially designed carious lesions, enabling more authentic caries excavation experiences for students [23].

Improvements to the tooth morphology (*n* = 2), the inclusion of a pulp chamber (*n* = 1), and the incorporation of a deeper core build-up (*n* = 1) were recommended improvements by the students. While these modifications could be considered for future iterations of the hands-on training course, it is important to note that the increased complexity of the training materials would entail greater preparation efforts. Complex designs of 3D printed teeth - comprising multiple components and differentiated by color - have been reported in various educational contexts, including endodontic training, teeth with integrated adhesive bridge preparation guides, and modular models for restorative and prosthetic dentistry [11, 17, 24].

The realism of the pre-prosthetic exercise using 3D printed teeth was rated for caries excavation at ⌀30.58 (± 24.74) and for the placement of the core build-up at ⌀47.77 (± 26.22). The tactile sensation during preparation was rated comparably between the 3D printed teeth and typodont teeth. Despite frequent student feedback requesting greater hardness of the 3D printed teeth (*n* = 43) and the known material limitations compared to natural tooth structures, the comparable tactile ratings between groups indicate a perceived equivalence in this aspect. The learning process, learning success, and overall suitability were rated with no statistical difference for both 3D printed and typodont teeth. Notably, these ratings for the 3D printed teeth showed a partial but significant increase from H1 to H2 and H3. Students predominantly expressed a desire for additional practice exercises using 3D printed teeth (⌀64.80) and indicated moderate openness to complete the entire training program exclusively with 3D printed materials to prepare for patient treatment (⌀47.84).

Overall, students provided valuable insights into the strengths and limitations of the 3D printed training teeth. While aspects such as anatomical accuracy and training flexibility were positively received, material properties - including hardness, surface texture, and component retention - require further optimization to fully support realistic and effective simulation in dental education.

### Educational implications

The use of 3D printing in dental education has generally been well received by students and provides educators with increased flexibility and variety in their teaching methods [12]. The novelty of this study is the context of bridge preparation with an integrated workflow encompassing caries excavation, core build-ups and fabrication of temporary restorations.

Karagkounaki et al. reported in their review on previous studies in dental education focusing on a single aspect of training as well as modular training models [12]. Other studies reported more pronounced benefits of 3D printed training materials. However, it must be emphasized that the concept applied in the present study was novel and more complex in its design. It could be argued that the complexity of the required skills and workflows may have exceeded the learners’ current routines. In previous preclinical practices students focused on individual steps without an integrated workflow. This interpretation aligns with the cognitive load theory in the context of medical education [25]. The transition from preclinical to clinical practice poses a significant challenge for students, necessitating tailored curriculum adjustments [4]. In clinical practice, integrated workflows encompassing pre-prosthetic and prosthetic procedures are standard and essential for patient care. This learning gap was the focus of this study, as previous course supervision revealed that students encountered difficulties when transitioning to these clinical workflows.

The 3D printed teeth from H1 provided the most comprehensive and realistic representation of an integrated workflow. Especially regarding the newly implemented changes the licensing regulations for dental education in Germany resulting in time constraints and the need for adaptive and wide-ranging training, these comprehensive and realistic training opportunities subsequently become more relevant. In fact, long-term educational outcomes and potential enhancements in patient treatment during clinical training constitute more meaningful endpoints than immediate student evaluations following the exercise. It is important to acknowledge that the complexity of the simulated treatment scenarios may have led to cognitive overload among students, particularly in H1. This form of simulation - featuring an integrated clinical workflow combining multiple procedural steps - was a novel concept in the context of their training. Previously, students had primarily practiced individual, isolated skills in structured exercises. The unfamiliarity with managing a realistic, patient-like workflow in a single session likely contributed to initial challenges and lower evaluations. Nevertheless, these integrated simulations are critical for developing clinical competence and bridging the gap between pre-clinical and clinical training. The adaptability of 3D printed exercises enables educators to tailor training scenarios to specific learning objectives or curriculum milestones. This flexibility supports competency-based education by allowing for the creation of progressive, patient-relevant cases that evolve with students’ skill levels. As dental education continues to emphasize clinical preparedness and critical thinking, 3D printed models offer a promising avenue for developing both procedural accuracy and decision-making in a safe, simulated environment. Standardized printed models can ensure uniformity in training conditions across cohorts and institutions, improving comparability and enabling objective benchmarking of performance.

Lee et al. introduced a simulated training approach using 3D printed, patient-specific models to support the transition from preclinical to clinical education, with the aim of better preparing students for their initial clinical experiences [26]. They emphasized the value of simulation as a transitional educational tool, particularly in the context of restorative procedures involving irreversible tooth preparations. The present study builds on this concept by employing 3D printed teeth for bridge preparation within an integrated workflow. This allows students to engage in realistic, patient-like, and logically structured practice sessions without performing invasive procedures or risking patient harm.

The total cost of resin was relatively low at €37.84. In contrast, expenses for typodont teeth used in bridge preparation training for 116 students amounted to €696. However, these typodont teeth do not support integrated workflows or allow for individual features such as core build-ups or customized tooth anatomies. The cost advantage of 3D printed teeth thus presents a significant benefit in dental education, offering a scalable and feasible solution. The 3D printed teeth developed in this study are feasible, allow for further customization, and could be produced in larger quantities. Richter et al. highlighted for their study with 3D printed dental models in undergraduate conservative training for caries excavation exceptional cost-efficiencies [27]. Nevertheless, primary costs associated with 3D printed teeth lie in the time and labor required for design, printing, post-curing, and assembly. Di Lorenzo et al. reported in their study with 3D printed teeth for endodontic training also on these associated indirect costs to the overall cost-efficient 3D printed training utilities [11]. Hands-on courses are accompanied by time-consuming and intense support and supervision by trained staff [28]. This, however, reflects authentic circumstances typical of educational environments.

Overall, the 3D printed teeth represented a valuable tool for students when training bridge preparations with defect-oriented preparation techniques, especially as typodont teeth offer only limited scope for customization, integrated workflows and complex preparation techniques. The null- hypothesis can thus be rejected.

## Conclusions

The 3D printed teeth enabled students to practice bridge preparations using defect-oriented techniques, encompassing various complexities such as caries removal, core build-ups, and provisional bridge fabrication. Although the data were primarily subjective and methodological limitations are acknowledged, the 3D printed teeth demonstrate potential as a valuable tool to enhance dental education.

## Data Availability

The datasets used and/or analyzed during the current study are available from the corresponding author on reasonable request.

## Abbreviations

H: Hands-on course
Q: Question
VAS: Visual Analog Scale
p: Statistical significance
r: Effect size
ε2: Effect size
1-β: Power

## References

[1] Juloski J, Radovic I, Goracci C, Vulicevic ZR, Ferrari M. Ferrule effect: a literature review. J Endod. 2012;38(1):11–9. 22152612 10.1016/j.joen.2011.09.024

[2] Naumann M, Schmitter M, Frankenberger R, Krastl G. Ferrule comes first. Post is second! Fake news and alternative facts?? A systematic review. J Endod. 2018;44(2):212–9. 29229457 10.1016/j.joen.2017.09.020

[3] Edelhoff D, Sorensen JA. Tooth structure removal associated with various Preparation designs for anterior teeth. J Prosthet Dent. 2002;87(5):503–9. 12070513 10.1067/mpr.2002.124094

[4] Jazzar A, Gadi R, Rajeh M, AlDehlawi H, Alhamed S, Badeeb T. Dental students’ perspective of transitioning from Pre-Clinical to clinical practice. Adv Med Educ Pract. 2024;15:1271–83. 39734781 10.2147/AMEP.S482341 PMC11675290

[5] Kessler A, Hickel R, Reymus M. 3D printing in Dentistry-State of the Art. Oper Dent. 2020;45(1):30–40. 31172871 10.2341/18-229-L

[6] Tomášik J, Zsoldos M, Oravcová Ľ, Lifková M, Pavleová G, Strunga M, Thurzo A. AI and Face-Driven orthodontics: A scoping review of digital advances in diagnosis and treatment planning. AI Vol. 2024;5:158–76.

[7] Tian Y, Chen C, Xu X, Wang J, Hou X, Li K, Lu X, Shi H, Lee ES, Jiang HB. A Review of 3D Printing in Dentistry: Technologies, Affecting Factors, and Applications. Scanning. 2021;2021:9950131. 34367410 10.1155/2021/9950131 PMC8313360

[8] Lepišová M, Tomášik J, Oravcová Ľ, Thurzo A. Three-Dimensional-Printed elements based on polymer and composite materials in dentistry: A narrative review. Bratisl Med J. 2025;126(1):14–27.

[9] Del Hougne M, Di Lorenzo I, Höhne C, Schmitter M. A retrospective cohort study on 3D printed temporary crowns. Sci Rep. 2024;14(1):17295. 39068274 10.1038/s41598-024-68354-2 PMC11283549

[10] Duan M, Lv S, Fan B, Fan W. Effect of 3D printed teeth and virtual simulation system on the pre-clinical access cavity Preparation training of senior dental undergraduates. BMC Med Educ. 2024;24(1):913. 39180072 10.1186/s12909-024-05869-2 PMC11344365

[11] Di Lorenzo I, Del Hougne M, Krastl G, Schmitter M, Höhne C. 3D printed tooth for endodontic training in dental education. Sci Rep. 2025;15(1):20185. 40542049 10.1038/s41598-025-06081-y PMC12181308

[12] Karagkounaki A, Manoukakis T, Margariti I, Pavlou C, Hadjichristou C. 3D printing in dental education: A review of its use across disciplines. Advance online publication. J Dent Educ 2025:e13876. 10.1002/jdd.13876. 10.1002/jdd.13876 40069932

[13] Model Resin Technical Data Sheet. [https://formlabs-media.formlabs.com/datasheets/2101617-TDS-ENUS-0.pdf]

[14] Plotino G, Grande NM, Bedini R, Pameijer CH, Somma F. Flexural properties of endodontic posts and human root dentin. Dent Mater. 2007;23(9):1129–35. 17116326 10.1016/j.dental.2006.06.047

[15] Rigid 10K Resin. Resin Technical Data Sheet [https://media.formlabs.com/m/522eb50e6c8ac0e3/original/-ENUS-Rigid-10K-TDS.pdf]

[16] Cresswell-Boyes AJ, Davis GR, Barber AH, Krishnamoorthy M, Nehete SR. An evaluation by dental clinicians of cutting characteristics and haptic perceptions in 3D-printed typodont teeth: A pilot study. J Dent Educ. 2025;89(4):567–77. 39444145 10.1002/jdd.13749 PMC12004343

[17] Höhne C, Del Hougne M, Gärtner L, Winter A, Schmitter M. Modular training model for education of students in restorative and prosthodontic dentistry. Eur J Dent Educ. 2024;28(1):347–57. 37804044 10.1111/eje.12956

[18] Kröger E, Dekiff M, Dirksen D. 3D printed simulation models based on real patient situations for hands-on practice. Eur J Dent Educ. 2017;21(4):e119–25. 27470072 10.1111/eje.12229

[19] Marty M, Broutin A, Vergnes JN, Vaysse F. Comparison of student’s perceptions between 3D printed models versus series models in paediatric dentistry hands-on session. Eur J Dent Educ. 2019;23(1):68–72. 30383320 10.1111/eje.12404

[20] Regish KM, Sharma D, Prithviraj DR. Techniques of fabrication of provisional restoration: an overview. Int J Dent. 2011;2011:134659. 22013441 10.1155/2011/134659 PMC3195530

[21] Lim NK, Shin SY. Bonding of conventional provisional resin to 3D printed resin: the role of surface treatments and type of repair resins. J Adv Prosthodont. 2020;12(5):322–8. 33149854 10.4047/jap.2020.12.5.322 PMC7604236

[22] Taokhampu N, Lekatana H, Palasuk J. Bond strength of aged provisional 3D-printed methacrylate resin with different surface treatments and repair materials. BMC Oral Health. 2025;25(1):789. 40413501 10.1186/s12903-025-06201-0 PMC12103029

[23] Carnier L, Del Hougne M, Schmitter M, Höhne C. 3D-printed tooth for caries excavation. BMC Med Educ. 2024;24(1):1243. 39482738 10.1186/s12909-024-06230-3 PMC11529318

[24] Del Hougne M, Behr G, Schmitter M, Höhne C. 3D printed teeth with adhesive Bridge Preparation guide. Sci Rep. 2024;14(1):22017. 39317710 10.1038/s41598-024-73433-5 PMC11422489

[25] Young JQ, Van Merrienboer J, Durning S, Ten Cate O. Cognitive load theory: implications for medical education: AMEE guide 86. Med Teach. 2014;36(5):371–84. 24593808 10.3109/0142159X.2014.889290

[26] Lee B, Kim JE, Shin SH, Kim JH, Park JM, Kim KY, Kim SY, Shim JS. Dental students’ perceptions on a simulated practice using patient-based customised typodonts during the transition from preclinical to clinical education. Eur J Dent Educ. 2022;26(1):55–65. 33512776 10.1111/eje.12672

[27] Richter M, Peter T, Rüttermann S, Sader R, Seifert LB. 3D printed versus commercial models in undergraduate Conservative dentistry training. Eur J Dent Educ. 2022;26(3):643–51. 34923733 10.1111/eje.12742

[28] Schwindling FS, Deisenhofer UK, Porsche M, Rammelsberg P, Kappel S, Stober T. Establishing CAD/CAM in preclinical dental education: evaluation of a Hands-On module. J Dent Educ. 2015;79(10):1215–21. 26427781

